# Ion Channel Activity of Vpu Proteins Is Conserved throughout Evolution of HIV-1 and SIV

**DOI:** 10.3390/v8120325

**Published:** 2016-12-01

**Authors:** Timo Greiner, Sebastian Bolduan, Brigitte Hertel, Christine Groß, Kay Hamacher, Ulrich Schubert, Anna Moroni, Gerhard Thiel

**Affiliations:** 1 Membrane Biophysics, Technische Universität Darmstadt, 64287 Darmstadt, Germany; timogreiner@googlemail.com (T.G.); Hertelb@bio.tu-darmstadt.de (B.H.); 2 Institute of Virology, Helmholtz Zentrum Munich, 85764 Oberschleißheim, Germany; sebastian.bolduan@gmx.de; 3 Computational Biology & Simulation Group, Deparment of Biology, Technische Universität Darmstadt, 64287 Darmstadt, Germany; c.gross@bio.tu-darmstadt.de (C.G.); Hamacher@bio.tu-darmstadt.de (K.H.); 4 Institute of Virology, Friedrich Alexander University, 91054 Erlangen, Germany; Ulrich.Schubert@viro.med.uni-erlangen.de; 5 Department of Biology and CNR IBF-Mi, Università degli Studi di Milano, 20122 Milano, Italy; Anna.moroni@unimi.it

**Keywords:** Vpu channel function, viroporin, virus channel evolution, Vpu transmembrane domain

## Abstract

The human immunodeficiency virus type 1 (HIV-1) protein Vpu is encoded exclusively by HIV-1 and related simian immunodeficiency viruses (SIVs). The transmembrane domain of the protein has dual functions: it counteracts the human restriction factor tetherin and forms a cation channel. Since these two functions are causally unrelated it remains unclear whether the channel activity has any relevance for viral release and replication. Here we examine structure and function correlates of different Vpu homologs from HIV-1 and SIV to understand if ion channel activity is an evolutionary conserved property of Vpu proteins. An electrophysiological testing of Vpus from different HIV-1 groups (N and P) and SIVs from chimpanzees (SIV_cpz_), and greater spot-nosed monkeys (SIV_gsn_) showed that they all generate channel activity in HEK293T cells. This implies a robust and evolutionary conserved channel activity and suggests that cation conductance may also have a conserved functional significance.

## 1. Introduction

The human immunodeficiency virus type 1 (HIV-1) accessory protein Vpu is a 15–20 kDa oligomeric type 1 integral membrane phosphoprotein [1,2,3], which is encoded exclusively by HIV-1 and related simian immunodeficiency viruses (SIVs), but not by the majority of SIVs and HIV-2. It has been shown that Vpu augments virus release by counteracting the human host cell restriction factor tetherin [4,5]. Moreover, Vpu has been shown to induce degradation of the CD4 receptor by the endoplasmic reticulum (ER)-associated protein degradation (ERAD) pathway [6,7,8,9]. The cytoplasmic domain of Vpu contains a pair of serine residues (at positions 52 and 56), which are constitutively phosphorylated by the casein kinase 2 (CK-2) [10]. CK-2 mediated phosphorylation of the two serine residues is critical to induce CD4 degradation [11,12,13] and for the assembly of transmembrane domains as homo-oligomers [14]. Computational studies advocate a model according to which a putative ion conducting pore is formed by the transmembrane domains of monomers, which assemble in a dynamic manner in a pseudo symmetry axis [14].

Experimental studies have confirmed that the Vpu protein can indeed generate a weakly cation selective ion channel activity [15,16] with so far unknown biological function [15,16]. It was previously shown that ion channel activity is not required for down-regulation of tetherin from the cell surface [16,17]. This once again raised the question of whether ion channel function of Vpu has any functional significance [17,18]. However, it was recently shown that the novel antiviral drug BIT225 (*N*-carbamimidoyl-5-(1-methyl-1*H*-pyrazol-4-yl)-2-naphthamide) inhibits Vpu generated ion channel activity. This block of Vpu channel activity can be correlated with a BIT225 mediated inhibition of HIV-1 replication in myeloid dendritic cells suggesting that a block of channel activity could be used in therapy for limiting viral spread [19,20]. A recent double-blind, placebo-controlled, randomized clinical phase 1b/2a study in 21 HIV-1-infected antiretroviral therapy (ART)-naive subjects, has shown that BIT225 treatment can indeed significantly reduce the viral burden in myeloid lineage cells and the level of monocyte activation [21,22]. These results gave the first indications that ion channel activity might play an important role in myeloid cells.

The recent analysis of many Vpu sequences has highlighted a considerable variability in this protein, placing it among the most highly variable proteins in the HIV-1 proteome [23]. Further studies have shown that this structural variability is causally related to the ability of different Vpus in down regulating CD4 and bone marrow stromal cell antigen 2 (BST2) proteins. Here we want to extend this study on examining the relationship between Vpu polymorphism and ion channel activity. The key question is whether channel function has been evolutionary conserved or whether it is just an epiphenomenon of the Vpu protein from M type HIV-1. To address this question, we examine the electrical properties of Vpu proteins from related immunodeficiency virus isolates. Vpu genes were originally only found in HIV-1 and SIVs from chimpanzees (SIV_cpz)_ but not in HIV-2. It is now well established that Vpus are also present in SIVs originating from other primates. The evolution of the Vpu proteins presumably reflects the evolution of SIV and HIV viruses. A detailed phylogenetic analysis of SIV/HIV [24] suggests that SIV_cpz_ rose to the pandemic (M, main) and non-pandemic (O, outlier and N, non-M, non O) groups of HIV-1; also the related SIV`s from gorillas (SIV_gor_) and the closely related and only recently detected HIV-1 group P, which can be traced back to the same origin. The SIV_cpz_ itself is presumably a product of a series of cross-species transmission and recombination events, which involved precursors of today’s SIV from various monkeys, namely the greater spot-nosed (SIV_gsn_), mona (SIV_mon_), Dent’s mona (SIV_den_), and Mustached (SIV_mus_) monkey. Hence, it is reasonable to assume that all *vpu* genes originate from a common ancestor of the SIV_gsn/mus/mon/den_ linage of primate lentiviruses [24].

After analyzing, in a previous study, the conductive properties of a Vpu protein from an M type HIV-1 [17] we analyze here the ion channel activities of different Vpu proteins of HIV-1 and SIV. The Vpu proteins represent different HIV-1 groups (N, P) and SIV_cpz_ and SIV_gsn_ [25,26]. Heterologous expression and electrophysiological characterization of the Vpu homologs clearly showed that all Vpus investigated generate comparable ion channel function in HEK293T cells. The results of these experiments imply a robust and evolutionary conserved ion channel activity, which suggests that a cation conductance may also have a conserved functional significance.

## 2. Materials and Methods

### 2.1. Bioinformatics:

For information theoretic calculations, we used a multiple sequence alignment of the Vpu protein family from the PFAM database (PFAM id PF00558) [27]. The full alignment contained 9232 sequences with an overall length of 168 positions including gaps. 9084 of these sequences are assigned to HIV whereas 23 sequences are assigned to the simian variant SIV. To investigate the conservation state of several residues in HIV Vpu, the HIV sequences of the alignment were further processed and used for the calculation of Shannon entropy. First, sequences with letters that do not encode natural amino acids (X, Y, Z, B, O, U) as well as sequences with less than 50 residues were deleted. Furthermore, positions with a gap content bigger than 60% were also deleted. This resulted in an alignment of 6947 sequences with 80 positions. Shannon entropy was calculated using the R package BioPhysConnectoR (version 1.6-10) [28]. The sequences of SIV Vpu were extracted, realigned with Clustal W (version 1.6-10), and processed the same way.

Selected Vpu sequences from HIVs and SIVs, which were previously examined for their ability to antagonize tetherin function [25], were aligned with Clustal W [29] using default parameters and afterwards manually optimized in Jalview (version 2.0) [30].

### 2.2. Heterologous Expression of Vpus:

Human embryonic kidney 293 T cells (HEK293T) (DSMZ, Braunschweig, Germany) were grown at 37 °C in a humidified 95% air/5% CO_2_ incubator in Dulbecco’s Modified Eagle Medium (DMEM; SIGMA-Aldrich, Taufkirchen, Germany) supplemented with 10% v/v heat-inactivated fetal bovine serum, 100 U/mL penicillin G and 100 μg/mL streptomycin sulfate (all from Invitrogen, Carlsbad, CA, USA). Cells were passaged after reaching 70%–80% confluence every 2–3 days.

Vpu variants from HIVs and SIVs, which were extensively described before [25,26], were a generous gift from Dr. Sauter (University of Ulm, Ulm, Germany). The Vpu genes were inserted as reported previously [25,26] in the pCG-IRES-GFP vector, which is expressing AU1-tagged Vpu together with green fluorescent protein (GFP) from bicistronic mRNA.

For expression, HEK293T cells were transiently transfected with aforementioned bicistronic vector using the liposomal transfection reagent TurboFect™ (Fermentas, Waltham, MA, USA). For all constructs we generally found a transfection efficiency between 10% and 20% judging from the green fluorescence (Figure S1). Twenty four hours post transfection cells were washed with phosphate-buffered saline (PBS), dispersed with trypsin (SIGMA-Aldrich) and seeded into new culture dishes with lower density. For patch clamp recordings, only isolated and adherent cells were considered. This ensures that recordings are from intact cells and that the currents reflect the conductance of a single cell of interest.

The expression of AU1 tagged Vpu proteins in HEK293T cells was tested by Western blotting. HEK293T cells were 48 h after transfection pelleted and subsequently lyzed. Vpu was detected by a monoclonal AU1 antibody (Covance, Munich, Germany); an anti-mouse immunoglobulin (Ig)-alkaline phosphatase antibody served as secondary antibody. Immune complexes were washed with wash buffer and separated in a 15% sodium dodecyl sulfate (SDS) gel.

### 2.3. Electrophysiological Characterization:

For the electrophysiological measurements, cells were transferred into a measuring chamber. The culture medium was subsequently removed and replaced by bath solution. The bath solution contained: 1.8 mM CaCl_2_, 1 mM MgCl_2_, 5 mM 4-(2-hydroxyethyl)-1-piperazineethanesulfonic acid (HEPES) and 50 mM KCl. The pH was adjusted to 7.4 with KOH. The osmolarity was adjusted to 330 mOsmol with mannitol. The pipette solution contained: 130 mM D-potassium-gluconic acid, 10 mM NaCl, 5 mM HEPES, 0.1 mM guanosine triphosphate (Na salt), 4 mM CaCl_2_, 2 mM MgCl_2_, 5 mM phosphocreatine, and 2 mM adenosine triphosphate (ATP, Na salt); the pH was adjusted to 7.4 with KOH, the osmolarity was adjusted to 330 mOsmol with mannitol. The measuring chamber was placed on an inverted epifluorescent microscope (Axiovert 100, Zeiss, Oberkochen, Germany) for patch clamp measurements. Cells were inspected under normal light for selecting isolated cells. Also, the fluorescence of cells was monitored by exiting GFP with blue light (390 ± 10 nm) from a monochromator (Till Photonics, Munich, Germany) and observing fluorescence >525 nm after passing the emitted light through a band pass filter (MF525-39, ThorLabs, Munich, Germany). Only green fluorescent cells were used for patch clamp measurements.

Whole-cell patch-clamp recordings were performed at room temperature using standard methods [31] with an EPC-9 patch-clamp amplifier (HEKA, Lamprecht, Germany). Cells were clamped from a holding voltage (0 mV) to test voltages between +80 and −160 mV and back to a post potential (−80 mV). The data were acquired and analyzed with the software (version 2*73, HEKA, Lamprecht, Germany). The number of cells, which were measured for each construct, is reported in Figure 3. Each construct was measured from ≥2 independent transfections.

## 3. Results

### 3.1. Sequence Variability of Vpu Proteins

**Figure 1:**
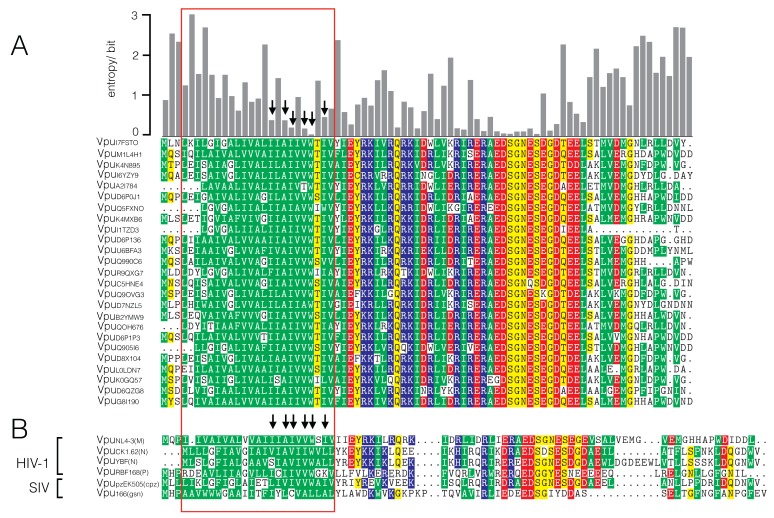
Sequence variability of Vpu proteins. (**A**) Alignment of the 25 most similar sequences out of 6947 sequences analyzed. Red frame indicates position of the transmembrane domain. Gray bars on the top give Shannon entropy for each column position from alignment of 6947 sequences. Arrows indicate amino acid positions with low entropy values (<0.5 bit) indicate strong conservation and hence high selective pressure among all known sequences; (**B**) Alignment of Vpu proteins of human immunodeficiency virus type 1 (HIV-1) and simian immunodeficiency virus (SIV), which were tested for channel function. They include Vpu proteins from different HIV-1 groups namely M group (VPUNL4-3 [AAB60577.1]), N group (VpuYBF30 [O91085.1], VpuCK1.62 [ACX46167.1]), and P group (VpuRBF168 [ACT66828.1]) as well as SIVs from chimpanzees (SIV_cpz_) [ABD19498.1] and greater spot-nosed monkeys (SIV_gsn_ [AAM90236.1]); the accession numbers for the Vpus are given in square brackets. Colors in alignment indicate acidic (red), basic (blue), polar uncharged (yellow), and hydrophobic nonpolar (green) amino acids.

At first, the sequence variability of Vpu proteins of HIV-1 and SIV was analyzed. For this purpose, we used the sequence of the HIV-1 NL4-3 Vpu [32], to browse for an alignment of the Vpu protein family within the PFAM database release 28.0 [27] and extracted 6947 sequences for an optimized alignment. Figure 1A shows the 25 most similar sequences. The gray bars on the top quantify the Shannon entropy for each position (column) of the full alignment. Since this entropy value is the average of variability contained in each column, low values indicate strong conservation of amino acids and high values indicate a greater variability—and thus less selective pressure on the respective position—among all known sequences.

The channel-forming region of the Vpu protein is in the N-terminal domain [33,34] between amino acid residues 4–26. In this region, we find in the alignment of 6947 sequences six positions with entropy values <0.5 bit; three of these have entropy values <0.25 bit. Interesting to note is that the Ser23, an amino acid, which was previously identified as crucial for channel function HIV-1 NL4-3 Vpu [17,34], is not among the highly conserved amino acids. Notably, some of the highly conserved positions are less conserved in the Vpu sequences from non-pandemic HIV strains (Figure 1B; Figure S2). It is possible that these deviations could be related to the functional differences of these Vpus. It was found that Vpus from the nonpandemic HIV-1 O strains exhibit only poor counteracting activity against tetherin while those from the rare group N viruses are not able to degrade CD4. From these data, it was deduced that only HIV-1 M evolved a fully functional Vpu during three independent cross-species transmissions [25,26].

Each of the selected sequences in Figure 1 differs at only few positions within the transmembrane domain (TMD) from the query sequence. Among all of the 6947 HIV sequences, the same amino acid substitutions can also be found in other Vpu homologs but with very low probability. In the Vpu homologs from SIVs however, the respective amino acids are more frequently found in the corresponding positions (Figure S1). The results of this analysis suggest that the high degree of conservation in parts of the TMD is not essential for the ion channel activity but may be required for other functional properties of Vpu. Table 1 shows the distribution (relative frequencies) of the most frequent amino acids of the highly-conserved positions of the HIV alignment—for SIV and HIV in comparison.

### 3.2. Various Vpu Proteins from Human Immunodeficiency Virus (HIV) and Simian Immunodeficiency Virus (SIV) Generate Channel Function

**Figure 2:**
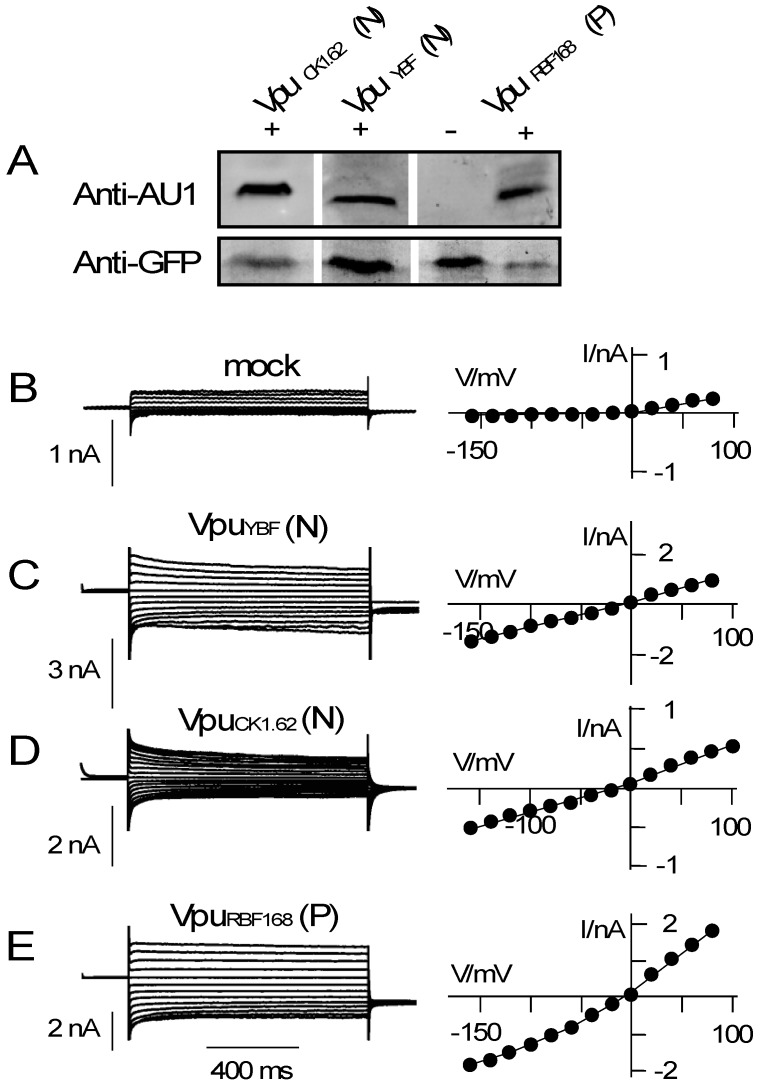
Conductive properties of Vpus from different HIVs. (**A**) Expression of different Vpu proteins in HEK293T cells was analyzed after cell lyses by Western blot, using anti-AU1 antibody. Successful transfection of cells was verified by anti-GFP antibody. The three Vpu proteins of interest comprised an AU1 tag (+). VpuRBF168 without AU1 tag was used as negative control (−). Example current responses (**left**) and corresponding I/V relations (**right**) of HEK293T cells transfected with either GFP alone (mock **B**) or with bicistronic vector for GFP plus VpuYBF (**C**), VpuCK1.62 (**D**), or VpuRBF168 (**E**). Letter in brackets indicates group of HIV-1 virus from which Vpu originates.

Against the background of apparent sequence variability among Vpus (Figure 1) and an apparent sensitivity of channel function to the fold of the transmembrane domain [17,34] we examined the channel activities of Vpus from HIV-1 and SIV. Candidate Vpu proteins were expressed in HEK293T cells and their electrical activity measured in positively transfected—i.e., green fluorescent—cells by patch clamp. The data in Figure 2A confirm that all Vpu constructs were indeed expressed in HEK293T cells. The level of expression among the different constructs was very variable between different experiments. Since we also found different efficiencies in transfection between different experiments and between different constructs, it remains unclear whether the constructs of interest have different levels of expression in HEK293T cells.

As a reference, we first monitored the currents of control cells, which were mock-transfected with GFP only. Figure 2B shows the current responses of a typical control cell to voltage steps from a holding voltage at 0 mV to test voltages between +80 mV and −160 mV in a bath solution with 50 mM KCl. The recorded currents and the corresponding current/voltage (I/V) relation shown in Figure 2B are characteristic for HEK293T cells [35]. They show very small negative currents and only slightly larger positive currents.

**Figure 3:**
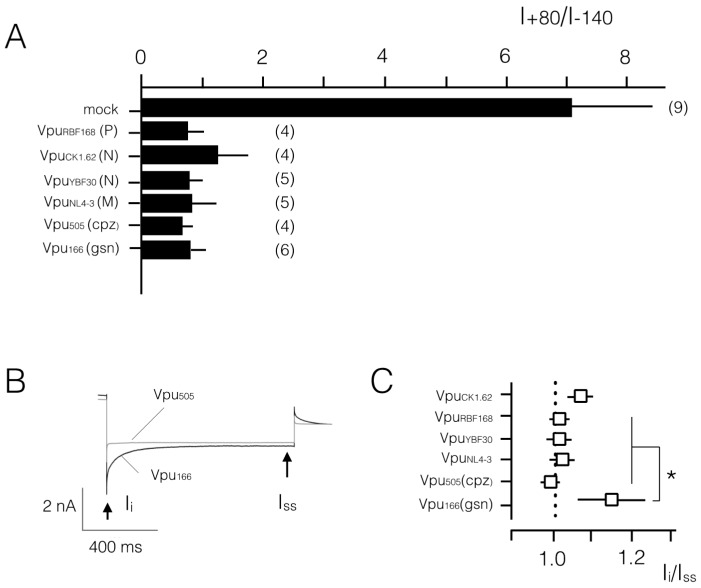
Analysis of current/voltage properties of Vpus from different HIVs and SIVs. (**A**) Mean ratios (I_+80_/I_−140_) with standard deviations (SD) of currents at +80 mV versus currents at −140 mV of mock transfected HEK293T cells and cells transfected with Vpu homologs. The high ratio indicates that mock-transfected cells exhibit an outward rectifying I/V relation while a value around 1 shows that the expression of all Vpu homologs generates an approximately linear I/V relation; (**B**) representative current responses of HEK293T cells transfected with either Vpu_cpz_ or Vpu_gsn_ to voltage step from 0 mV to −160 mV. While the former exhibits no apparent time dependency, the latter decreases with time; (**C**) Kinetics of current responses to negative voltage steps in cells expressing different Vpus is quantified by ratio (I_i_/I_ss_) of current at start of voltage pulse (I_i_) divided by steady state current (I_ss_) at end of pulse. Numbers in brackets in **A** report the number of measured cells; the same data were used for the analysis in **B** and **C**. A Student's *t*-test shows that the data are different between mock transfected cells and Vpu expressing cells in **A** (*p* < 0.0005) and between indicated Vpu constructs and Vpu_166_(_gsn_) in **C** (*p* < 0.05, *). Data on the Vpu_NL4-3_ generated increase in membrane conductance in HEK293T cells are re-plotted from [17] for comparison with other Vpus from HIV1 or SIVs.

As a result of the low conductance at negative voltages, the I/V curve is hardly distinguishable from the voltage axis (Figure 2B). Because of an endogenous outward rectifying channel in these cells, the I/V relation exhibits a small slope at positive voltages. For further analysis, the I/V relations of control cells can be best quantified by the ratio of currents at +80 mV versus that at −140 mV (I_+80_/I_−140_). In *n* = 9 mock-transfected cells we measured under the same experimental conditions a mean ratio I_+80_/I_−140_ of 7.0 ± 1.4 (Figure 3A).

Currents of cells, which express a Vpu protein from non-pandemic HIV-1, namely of the N and P group, are different from those of control cells. Figure 2C–E shows representative current recordings and I/V curves of HEK293T cells, which were transfected with the respective HIV-1 Vpus. The transfected cells are typically exhibited in response to the same voltage clamp steps, which were also used for the controls, much larger positive and negative currents than the controls. While the absolute current density was variable between different cells and different constructs (e.g., Figure 2B,C) the additional currents rendered the I/V relations approximately linear over the window of test voltages (Figure 2C–E). The same overall increase in membrane currents with a near linear I/V relation was confirmed in other HEK293T cells, which express one of the aforementioned Vpus. The linearity of the I/V curve is reflected in the I_+80_/I_−140_ ratio, which is in average for all Vpus ≤1.2 (Figure 3A). A comparison of the present results with similar measurements in which a Vpu from an M group HIV-1 (Vpu_NL4-3_) was expressed in HEK293T cells exhibits no appreciable differences. Also, Vpu_NL4-3_ generates an elevated inward current with a near linear I/V relation after heterologous expression in HEK293T cells (Figure 3A [17]).

Our data show that all Vpu proteins of HIV-1 generated irrespective of the sequence variations in the transmembrane domain a current. These currents must reflect a channel activity of the tested Vpus in the plasma membrane of the HEK293T cells. This interpretation is supported by the fact that a Vpu mutant, which contains a fully randomized TM sequence or a critical single point mutation, do not generate any currents in HEK293T cells [17]. Furthermore, there are to the best of our knowledge no endogenous currents in HEK293T cells, which resemble those recorded in cells expressing different Vpus [36]. In particular, the TASK channel, which was suggested as an interaction partner of Vpu [37] is an outward rectifier [38] and not voltage independent like those recorded here. Finally, the Vpu generated conductance in HEK293T cells, which is shown in Figure 3, is also in good agreement with measurements in *Xenopus* oocytes. In this alternative expression system Vpu generates a similar conductance with a quasi-linear I/V relation [39]. The diversity of endogenous channels in HEK293T cells and *Xenopus* oocytes [36,40] makes it unlikely that the conductances in Vpu expressing cells are caused by an upregulation of endogenous channels.

**Figure 4:**
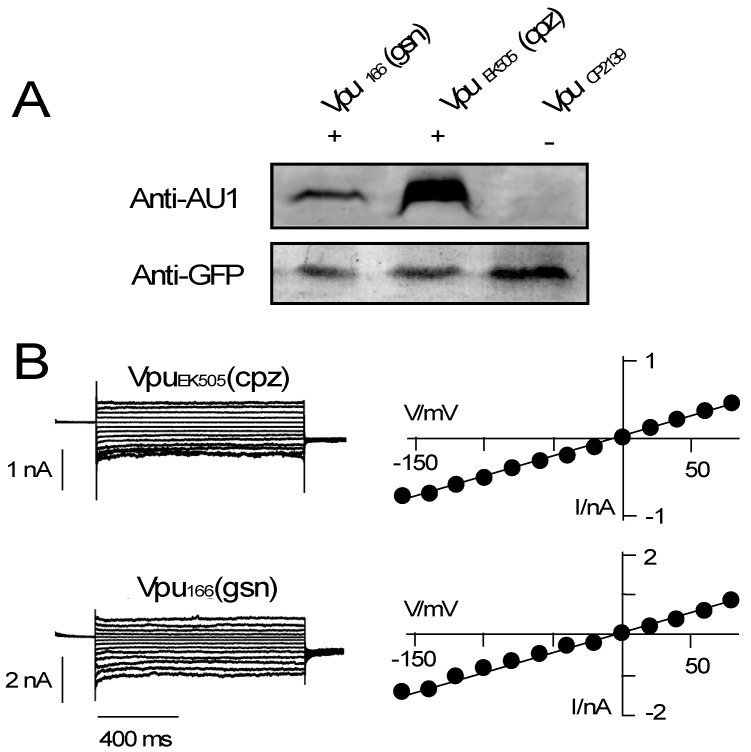
Conductive properties of Vpus from two SIVs. (**A**) Expression of different Vpu proteins in HEK293T cells was analyzed by anti-AU1 and anti-GFP antibodies as in Figure 1. The Vpu proteins of interest (VpuEK505, Vpu166) comprised an AU1 tag (+). VpuCP2139 without AU1 tag was used as negative control (−); (**B**) Example current responses (**left** panel) and corresponding I/V relations (**right** panel) of HEK293T cells transfected with either, Vpu_EK505_ from chimpanzee (cpz), or Vpu_CK1.62_ from greater spot-nosed monkey (gsn).

The apparent channel activity of different Vpus from HIV-1 and the phylogenetic relationship between HIV and SIV further suggest that also the Vpus from SIVs should generate a channel conductance. To test this hypothesis, we expressed Vpus from related SIVs namely, SIV_cpz_ and SIV_gsn,_ in HEK 293T cells (Figure 4). In terms of evolution, the latter can be considered the oldest Vpu allele among those considered here [41].

The Western blots in Figure 4A demonstrate that the Vpus from the two SIVs were indeed expressed in HEK293T cells. In the electrophysiological assay both Vpu proteins again generated a significant inward current (Figure 4B), which was larger than that measured in control HEK293T cells (Figure 2A). A plot of the mean I_+80_/I_−140_ ratios shows that also the Vpus from the primate SIVs generated a quasi-linear I/V relation in HEK293T cells (Figure 3A). Both Vpus from SIV_cpz_ and SIV_gsn_ generate in HEK293T cells roughly the same current density (Figure 4C).

Currents from all Vpus showed an overall similar phenotype but with some subtle variations. One subtle difference between the currents is visible in their kinetics. A scrutiny of the current responses shows in some cases a slight current inactivation at negative voltages (e.g., Figure 2C,D and Figure 4B). This is not consistent among all cells transfected with the same Vpu variant though (Figure 3B). Only in the case of the Vpu_gsn_ protein we find a robust difference between the initial (I_i_) and steady state current (I_ss_) (Figure 3B). These data imply that the respective Vpu_gsn_ undergoes an inherent inactivation at negative voltages.

## 4. Discussion

The key question of the present study was to examine whether the ion channel function, which has been found for the Vpu from M type HIV-1 [16,17], has been conserved through the evolution of this protein. The data show that Vpu proteins from different origins contain some highly conserved amino acid positions in the transmembrane domain, e.g., the domain, which is responsible for channel formation. These amino acids are not fully conserved in Vpu homologs from non-pandemic HIVs or Vpus from SIVs. The most highly conserved Trp23 for example is substituted by a Leu in Vpu_gsn_. A functional testing of the homologs however shows that all the tested Vpu homologs generated a current in HEK293T cells; channel activity is maintained irrespective of sequence variations in the TMD. Already Vpu_gsn_, the evolutionary oldest of the proteins tested here [41], generates channel function. Collectively, this implies that channel formation is an inherent and evolutionary old property of the Vpu protein. Negative effect of mutations on channel function like an exchange of Ser in position 23 in VPU_NL4-3_ [17,34] must have been compensated by other mutations in the protein in order to maintain channel activity.

The present data, which were collected by overexpressing Vpus in a model mammalian cell, do not provide any answer on the functional significance of the channel activity of the Vpu proteins in virus replication. However, the fact that this function has been maintained throughout evolution of different HIV and SIV viruses implies an evolutionary pressure and hence a function of channel activity at one stage of replication.

While the data show that different Vpus function as channels, the sequence variations between the proteins are not entirely insignificant for channel function. They seem to affect in some cases the kinetics of the channel in a heterologous expression system. Apart from these small differences in the kinetics, the data show no striking difference between the Vpus from various HIVs and SIVs. In this sense, the present results also underscore previous data, which have shown that the ability of Vpu to degrade CD4 and antagonize tetherin is not connected to its ion channel function [17]. Here we show that ion channel activity is highly conserved among the Vpu proteins of HIV-1 and SIV and this function is independent of the previously tested ability of Vpu to counteract tetherin [25].

## Figures and Tables

**Table 1:** Most frequent amino acids at highly conserved positions in the transmembrane domain of Vpu from human immunodeficiency virus type 1 (HIV) and simian immunodeficiency virus (SIV). Data are given as relative frequencies in % of the respective alignment (HIV vs. SIV) *.

**Position**	17				19				20			22				23		25	
**HIV**	I				A				I			V				W		I	
	94.8				94.4				97.3			97.9				99.6		93.5	
**SIV**	I	L	A		V	A, N	T		I	V		V	I	A		W		K	I
	36.4	22.7	22.7		27.3	22.7	18.2		63.6	27.3		40.9	27.3	18.2		95.5		40.9	27.3

* Note, that in all positions an almost perfect conservation (>90%) in HIV is reduced to still noticeable abundance (~20–60%).

## Supplementary Materials

The following are available online at www.mdpi.com/1999-4915/8/12/325/s1, Figure S1: Transfection of HEK293T cells Vpu from HIV-1, Figure S2: Alignment of Vpu orthologs from SIV with consensus sequence of Vpu from HIV-1.
