# Supplementary material for: Ion Channel Activity of Vpu Proteins Is Conserved throughout Evolution of HIV-1 and SIV
Source: Viruses. 2016 Dec 1;8(12):325. doi: 10.3390/v8120325 (PMC5192386; doi:10.3390/v8120325)
Supplement: Supplementary file 1 [file viruses-08-00325-s001.docx]

Supplementary Materials: Ion Channel Activity of Vpu Proteins Is Conserved throughout Evolution of HIV-1 and SIV

Timo Greiner, Sebastian Bolduan, Brigitte Hertel, Christine Groß, Kay Hamacher,
Ulrich Schubert, Anna Moroni and Gerhard Thiel


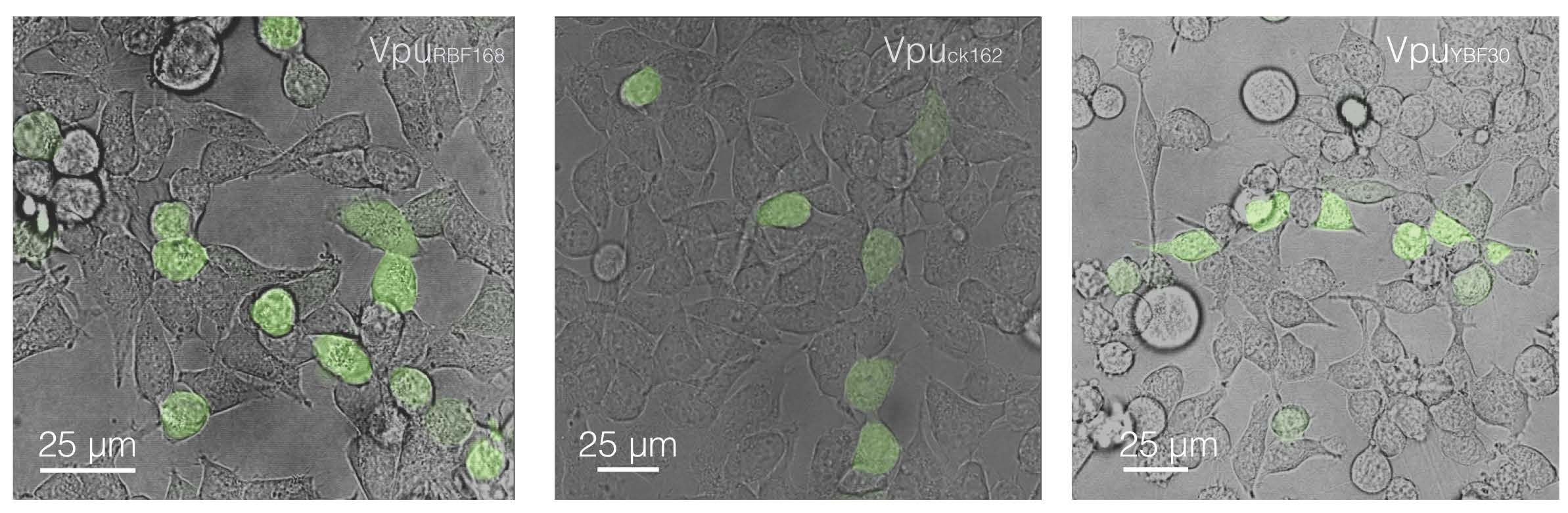


**Figure S1**. Transfection of HEK293T cells with Vpus from HIV-1. Exemplary images of HEK293T cells transiently transfected with bi-cistronic vector containing different Vpus (from left to right: VpuRBF168, VpuCK1.62, VpuYBF30) together with GFP. Images are overlays of bright field images revealing cell contours and fluorescent images showing positive GFP signal in individual cells.


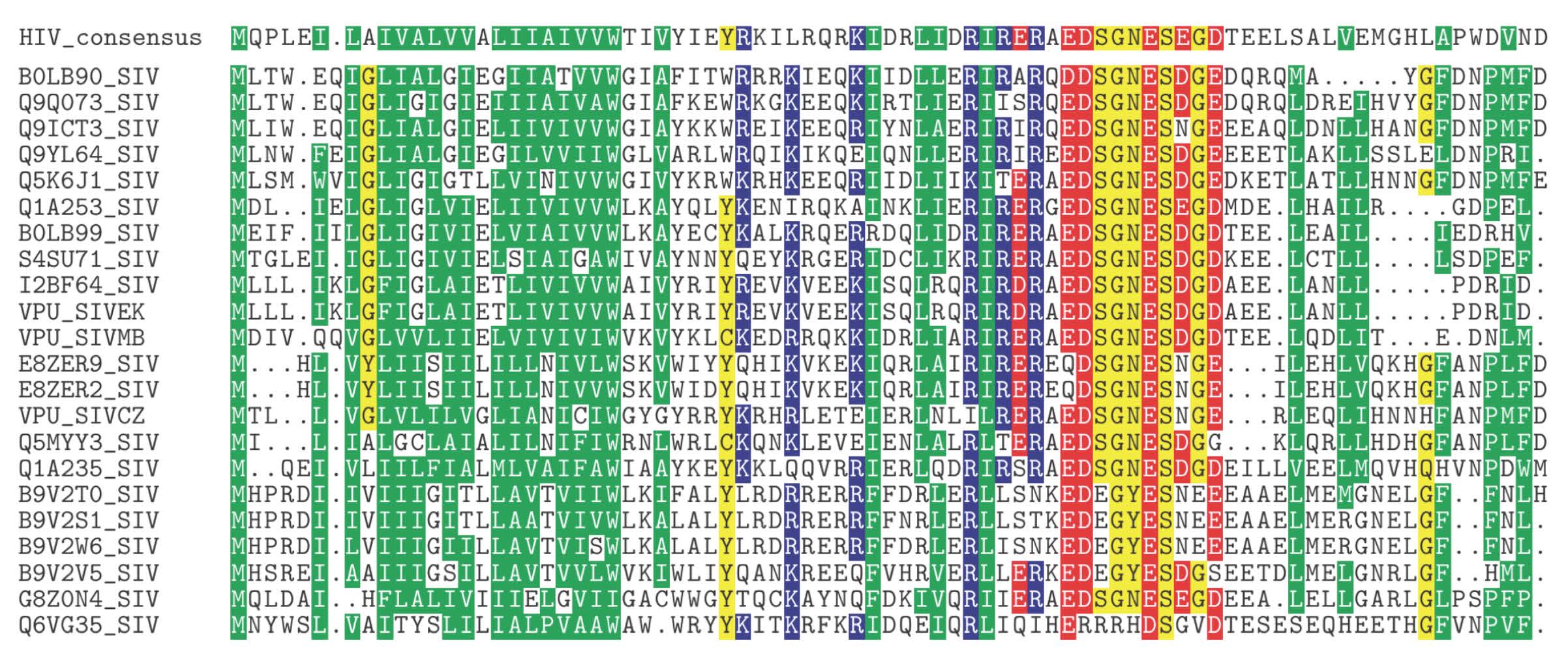


**Figure S2.** Alignment of Vpu orthologs from SIV with consensus sequence of Vpu from HIV-1. Consensus sequence (top lane) for Vpu protein was constructed from alignment of 6497 HIV Vpu entries in PFAM database. The SIV sequences within the PFAM alignment were extracted realigned amongst each other, and processed the same way as the HIV sequences. This resulted in the above alignment of 22 SIV sequences.

© 2016 by the authors; licensee MDPI, Basel, Switzerland. This article is an open access article distributed under the terms and conditions of the Creative Commons by Attribution (CC-BY) license (http://creativecommons.org/licenses/by/4.0/).
